# Detection of Zika virus infection among asymptomatic pregnant women in the North of Peru

**DOI:** 10.1186/s13104-018-3400-z

**Published:** 2018-05-18

**Authors:** Claudia Weilg, Lucinda Troyes, Zoila Villegas, Wilmer Silva-Caso, Fernando Mazulis, Ammy Febres, Mario Troyes, Miguel Angel Aguilar-Luis, Juana del Valle-Mendoza

**Affiliations:** 1 School of Medicine, Research and Innovation Centre of the Faculty of Health Sciences, Universidad Peruana de Ciencias Aplicadas, Av. San Marcos Cuadra 2, Chorrillos, Lima, Peru; 2 Laboratorio de Biología Molecular, Instituto de Investigación Nutricional, Lima, Peru; 3 Dirección Subregional de Salud de Jaén, Ministerio de Salud, Cajamarca, Peru; 4 Instituto de Investigación de Enfermedades Infecciosas, Lima, Peru

**Keywords:** Zika virus, Pregnant women, Outbreak of Zika, Peru

## Abstract

**Objective:**

To report an outbreak of ZIKV infection among asymptomatic pregnant women during 2016 in the city of Jaen, Cajamarca.

**Results:**

Zika virus RNA was detected in 3.2% (n = 36) of cases by RT-PCR. The mean age of patients positive for ZIKV infection was 29.6 years. 7 patients (19.4%) infected with ZIKV were in their first-trimester of gestation, 13 (36.1%) were in their second-trimester, and 16 (44%) were in their third-trimester. All of the infected pregnant women were asymptomatic. ZIKV infection remains a major public health issue that calls for constant epidemiological surveillance. It can cause the congenital Zika virus syndrome in the newborns of infected mothers. The lack of molecular diagnostic methods in isolated localities and the similarity of symptoms to other arboviral infections, lead to an under-diagnosis of this disease in endemic areas.

## Introduction

Zika virus (ZIKV) is an emergent arthropod-borne flavivirus associated with neurologic complications [1, 2]. The first reports of human disease were described in Uganda and Tanzania during an outbreak in 1952 [3, 4]. On February 2016, the World Health Organization (WHO) declared Zika virus infection a Public Health Emergency of International Concern (PHEIC) due to the increasing number of cases and its associated complications. Currently, ZIKV is responsible for the ongoing outbreaks in the Caribbean and South America following the initial outbreak in Brazil [5].

The primary route of human ZIKV transmission is through the bite of mosquitoes *Aedes aegypti* and *Aedes albopictus* in tropical and temperate regions [5–7]. ZIKV is also transmitted via transfusion of blood products, organ transplantation, laboratory exposure, sexual and, vertical maternal–fetal transmission [8–10]. After an incubation period of 2–14 days, patients develop an acute febrile syndrome associated with unspecific symptoms such as headaches, arthralgia, pruritic rash, nonpurulent conjunctivitis, myalgia, anorexia, asthenia, dysesthesia and retro-orbital pain [11, 12]. Despite the low frequency of fatalities and hospitalization due to severe disease, ZIKV infection is associated with a high frequency of neurologic complications such as congenital microcephaly [13] and Guillain–Barre syndrome [14]. ZIKV infection during pregnancy can cause a set of fetal developmental complications, known as the congenital Zika virus syndrome [15]. Along with this syndrome, ZIKV is also associated with a higher rate of pregnancy loss and stillbirths among infected women [16, 17].

In Peru, the first ZIKV infection reported was a Venezuelan immigrant in January of 2016 [18, 19]. On March of 2016, a middle-aged woman from Lima, infected with ZIKV via sexual transmission, became the first autochthonous case in Peru reported by the Ministry of Health [20]. In the latest epidemiological update submitted by the PAHO in August of 2017, they describe a decreasing trend in the number of cases confirmed in South America since March of 2016 [18]. In contrast, some Peruvian regions such as Cajamarca, Loreto, Ica, Tumbes, La Libertad, and Lima, 8080 cases have reported with an increasing trend of confirmed cases since 2016 [21, 22]. To date, no pregnancy complications or congenital microcephaly have been attributed to a ZIKV infection in Peru, according to the Peruvian Ministry of Health [22]. This study aims to describe an outbreak of ZIKV infection in a newly endemic region of Peru among asymptomatic pregnant women and evaluate newborn congenital complications in the city of Cajamarca in 2016.

## Main text

### Methods

#### Study setting

This study was carried out between May and July of 2016 in the province of Jaen. This town has a population of 135,021, is located in the North of Peru in the city of Cajamarca and is endemic to other arboviral diseases such as dengue. Cajamarca is one the poorest regions in Peru, with a 56% of its population living in poverty compared to the national average of 36% [23]. Cajamarca is also one of the regions with a higher incidence of maternal deaths with 31 cases reported a year prior this study in 2015 [24].

Venous and urine samples of 1116 asymptomatic pregnant patients, were collected in the Regional Laboratory of the Jaen Medical Health Network as part of the pregnancy screening program. The inclusion criteria were pregnant patients with a current or recent history of residence in Jaen. Pregnant women with acute undifferentiated febrile illness with symptoms such as headaches, muscle pain, ocular and/or joint pain, rash, nausea, asthenia, anorexia, or any other symptom that could suggest an infection.

Patients positive to ZIKV were included in a microcephaly protocol with ultrasound evaluation upon diagnosis and every three thereafter. The cephalic perimeter of the newborn was measured at birth and at 24 h. Microcephaly was defined as an occipitofrontal diameter greater than two standard deviations below the mean or less than the 3rd percentile based on standard growth charts for sex, age, and gestational age at birth [25]. Newborns were followed up 6 months later by a pediatric neurologist.

#### Ethics statements

Samples were collected following a written informed consent signed by all participants before enrollment or by the parents or respective guardians in the case of underage patients below 18 years of age. The study was approved by the Research Ethics Board of the *Hospital Docente Regional de Cajamarca* in Peru.

#### Samples

A venous blood sample was collected per patient by using Vacuette^®^ EDTA blood collection tubes (Greiner Bio-One GmbH, Frickenhausen, Germany) and these were stored at 4 °C until processing.

#### RNA extraction

The RNA was extracted from 200 µl of blood samples using High Pure Kit Preparation template (Roche Diagnostics GmbH, Mannheim, Germany).

#### Real-time RT-PCR assay for Detection ZIKV with taqman probe

A one-step RT-PCR was performed as described by Alva et al. [26]. The primers and the probe used are the ones described by Faye et al. [27].

### Results

1116 samples of pregnant women were studied from May to July 2016. None of the recruited samples were excluded from the data analysis due to exclusion criteria. Of all the samples, 3.2% (n = 36) of cases were positive for ZIKV by RT-PCR.

Of the positive cases for infection by ZIKV confirmed by RT–PCR, the mean age of patients was 29.6 years and 8.3% (n = 3) were below 18 years of age. In relation to the gestational age of the infected mothers, 7 patients (19.4%) were in the first trimester, 13 (36.1%) were in the second trimester and 16 (44%) were in their third trimester of pregnancy (Table 1).

**Table 1:** General characteristics of asymptompatic pregnant women

Characteristics	Total samples, n = 1116 (%)	Zika virus positive, n = 36 (%)
Age (years)		
< 18	84 (7.5)	3 (8.3)
18–35	896 (80.3)	31 (86.1)
> 35	136 (12.2)	2 (5.6)
Mean age		29.6
Place of residence		
Jaén	923 (82.7)	36.0 (100)
San Ignacio	190 (17.0)	0 (0)
Cutervo	3 (0.3)	0 (0)
Gestational age (weeks)		
1–12	222 (19.9)	7 (19.4)
13–27	460 (41.2)	13 (36.1)
> 27	434 (38.9)	16 (44.4)

All the ultrasound evaluations (n = 33) were negative for microcephaly, and the head circumference measured at birth and during the postnatal controls were within normal limits. According to the evaluation of normal growth and development of the newborns of the infected mothers, there was no anomaly found. Three infected pregnant women with a diagnosis during their third trimester were lost before the follow-up.

### Discussion

Zika virus infection has emerged as a major public health issue in the Americas. The broad geographical range of the vector and the emerging complications of the ZIKV infection have made this flavivirus a growing global concern that calls for constant epidemiological surveillance and a strategic response plan [28]. One of the target populations for the public health strategies are pregnant women because of the associated complications that ZIKV infection could have on them and their newborns [17]. Our findings show a prevalence of 3.2% (n = 36) of ZIKV infection among the asymptomatic pregnant women population of Jaen in the region of Cajamarca, which is higher than the 31 confirmed cases of ZIKV in asymptomatic women during 2016 in Peru [22]. The mentioned findings may indicate that there is an underestimation of ZIKV infection that could be explained by the lack of sensitive and specific molecular diagnostic methods in isolated localities. Furthermore, the prevalence in this study highlights the importance of epidemiological surveillance of ZIKV infection in pregnant women that live in newly ZIKV endemic regions such as Jaen. These findings cannot be extrapolated to the ZIKV infection rates among pregnant women in other endemic regions of Peru because of the lack of similar molecular diagnostic studies in other Peruvian localities. In South America, the incidence of ZIKV peaked in February 2016 and subsequently showed a declining tendency with the exception of Ecuador [18, 29] Peruvian data showed an opposite trend that may be due to an underestimation of the prevalence during 2016 caused by the lack of a consolidated surveillance program.

Congenital ZIKV syndrome may include microcephaly, ventriculomegaly, intracranial calcifications, extra-axial fluid, decreased brain parenchymal volume, lissencephaly, cerebellar hypoplasia, delayed myelination and hypoplasia of the corpus callosum [30–32]. Furthermore, newborns of infected mothers can develop cardiac anomalies with septum defects [33], hearing loss [34], seizures due to the underlying brain malformations, neuromotor abnormalities such as spasticity and feeding difficulties [15, 35], ocular abnormalities [36] and can be born small for the gestational age [37].

The most plausible ZIKV pathogenesis hypothesis of neurological complications describe that placental colonization and injury following maternal infection leads to the transmission of the virus to the fetal brain [38–40], where ZIKV preferentially infects neural progenitor cells [41]. As a result, this arbovirus disrupts neuronal growth, migration, proliferation, and differentiation, thus causing thinning of the cortex, macroscopic signs of microcephaly and impairment of normal brain development in the fetus [42]. This hypothesis also suggests the possibility that the placental inflammatory process contributes to the development of mentioned neurological complications [43].

The maternal age is also strongly associated with different pregnancy outcomes [44]. The risk of having a child with a congenital anomaly increases with advanced maternal age [45, 46], and it is not always related to the recognized increase in aneuploidy rate [47]. The US National Birth Defects Prevention Study found that newborns of women older than 40 years old had an increased risk of cardiac defects, craniosynostosis, esophageal atresia and hypospadias [47]. Also, studies consistently report that women older than 35 years old have a significantly increased risk of spontaneous abortion [48] and stillbirth compared to younger women [49]. ZIKV infection of pregnant women can also cause pregnancy losses and congenital malformations such as cardiac defects [33] and craniosynostosis [50], which may cause bias when estimating the risk of congenital ZIKV syndrome among women older than 35 years old. In our study, the mean age of infected pregnant women was 29.6 years and only 5.6% (n = 2) were older than 35 years.

The risk of vertical transmission and congenital ZIKV syndrome exists for both symptomatic and asymptomatic mothers [51, 52]. It is important to mention that only 20% of the patients positive for ZIKV infection are symptomatic [53, 54], which is why international guidelines recommend health care providers to screen all pregnant women for possible exposure to ZIKV at each prenatal visit, and to test those with ZIKV exposure risk three times during pregnancy even if they are asymptomatic [55].

The frequency of birth defects resulting from vertical transmission of ZIKV is not well established [56]. However, the risk of developing the congenital ZIKV syndrome is higher during the first and second trimester, even though severe fetal or newborn sequelae can also occur within the third-trimester infection [57, 58]. A prospective study in Brazil found that adverse outcomes were reported in 55% of pregnancies after first-trimester maternal infection, 52% after second-trimester maternal infection, and 29% after third-trimester maternal infection [17]. Our study found that most of the infected pregnant women (44%, n = 16) got ZIKV infection during the third trimester and that none of the newborns developed microcephaly. These findings are similar to those published by the Peruvian National Epidemiological Center for Control and Prevention of Diseases [59] and can explain why there are no reported cases of congenital ZIKV syndrome in Peru yet. Nonetheless, 19.4% (n = 7) and 36.1% (n = 13) of pregnant women were infected during their first and second trimester, respectively. This fact highlights the importance of prenatal ultrasonography to evaluate fetal abnormalities consistent with congenital ZIKV syndrome [60, 61], and the need of careful clinical evaluation of these infants in ruling out microcephaly, ocular abnormalities, hearing loss, neurologic and positional abnormalities, craniofacial disproportion or cardiac defects [62].

In conclusion, the disease caused by ZIKV is an emerging infection that remains a major public health issue in South America due to their endemicity in isolated localities. This study reports the first molecularly confirmed outbreak in the region of Cajamarca in Peru among asymptomatic pregnant women and describes a higher frequency of infection in comparison to the national surveillance report. The unavailability of molecular diagnostic methods in isolated localities and the similarity of symptoms to other arboviruses leads to an under-diagnosis of this infection in arboviral endemic regions therefore, it is molecular diagnosis of ZIKV infection in pregnancy screening programs to evaluate and measure the impact of fetal anomalies associated with this virus, especially amongst asymptomatic patients in endemic regions.

## Limitations

This study has two main limitations. First, the follow-up evaluation of the infant carried out by a pediatric neurologist, only occurred at 6 months of age. Second, there wasn’t a follow up protocol looking for other ZIKV associated malformations for the newborns of infected mothers.

## Abbreviations

ZIKV: Zika virus
RT-PCR: reverse transcription-polymerase chain reaction
RNA: ribonucleic acid
bp: base pairs
